# Gyre-driven decay of the Earth's magnetic dipole

**DOI:** 10.1038/ncomms10422

**Published:** 2016-01-27

**Authors:** Christopher C. Finlay, Julien Aubert, Nicolas Gillet

**Affiliations:** 1 Division of Geomagnetism, DTU Space, Technical University of Denmark, Lyngby DK-2800, Denmark; 2 Dynamique des Fluides Géologiques, Institut de Physique du Globe de Paris, 75238 Paris CEDEX 05, France; 3 ISTerre, Université Grenoble 1, CNRS, 38041 Grenoble CEDEX 9, France

## Abstract

Direct observations indicate that the magnitude of the Earth's magnetic axial dipole has decreased over the past 175 years; it is now 9% weaker than it was in 1840. Here we show how the rate of dipole decay may be controlled by a planetary-scale gyre in the liquid metal outer core. The gyre's meridional limbs on average transport normal polarity magnetic flux equatorward and reverse polarity flux poleward. Asymmetry in the geomagnetic field, due to the South Atlantic Anomaly, is essential to the proposed mechanism. We find that meridional flux advection accounts for the majority of the dipole decay since 1840, especially during times of rapid decline, with magnetic diffusion making an almost steady contribution generally of smaller magnitude. Based on the morphology of the present field, and the persistent nature of the gyre, the current episode of dipole decay looks set to continue, at least for the next few decades.

## Abstract (web summary)

The magnitude of the Earth's magnetic dipole has decreased by 9% over the past 175 years. Here, the authors suggest that the rate of dipole decay is controlled by a huge gyre in the liquid metal outer core acting on a field asymmetry, and that decay is set to continue for the next few decades.

## Graphical abstract

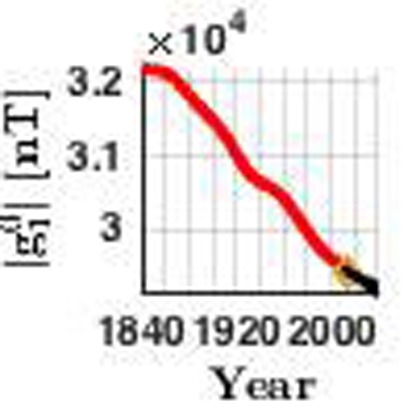

A long-standing problem in geophysics is the origin of the ongoing decay in the strength of the dipolar part of the Earth's magnetic field^1^,^2^,^3^. Direct measurements of the field intensity, available since the time of Gauss^4^,^5^, indicate that the dominant axial component of the dipole field, parallel to the planetary rotation axis, has been decreasing at a mean rate^6^ of 16 nT yr^−1^—see Fig. 1. The decay rate exhibits surprisingly large fluctuations on relatively short decadal time scales; the field was decaying twice as fast in 1980 as it is today. The physical process responsible for the dipole decay must, therefore, also evolve on fast decadal time scales. The Earth's magnetic field is generated by a dynamo operating within the liquid metal outer core. Here fluid motions stretch, twist, and transport magnetic field lines^7^,^8^,^9^ converting kinetic energy into magnetic energy, driving the evolution of the field and maintaining it against Ohmic dissipation. Improved understanding of the mechanism of dipole decay thus requires study of the motions taking place within the core and determining how these produce the observed diminishing.

The obvious explanation of a free Ohmic decay process, resulting from the finite electrical conductivity of the core, is untenable as it is about 20 times too slow. Free decay of the dipole would take ∼55,000 years based on the latest estimates of core conductivity^10^,^11^, whereas if the mean decay rate^6^ between 1840 and 2010 of 16 nT yr^−1^ were to continue, the axial dipole would reach zero within 1,900 years. Furthermore, free decay is incompatible with the accelerations in the rate of decay observed during the past 2,000 years^12^. Two alternative mechanisms, both driven by fluid motions within the core, have therefore been proposed. The first is the growth by magnetic diffusion of reversed flux features at the core–mantle boundary via toroidal flux expulsion^2^,^13^. It is, however, difficult to conclusively demonstrate that growth of reversed flux patches is occurring at the rate required to explain the observed dipole decay and its fluctuations^14^. A second possibility is that flow in the core acts, on average, to transport normal magnetic flux towards the equator^12^ and reversed flux poleward^2^,^15^. This meridional flux advection mechanism operates even in the absence of magnetic diffusion, and involves the transfer of magnetic energy from the axial dipole to other field components, rather than a direct loss to heat via Ohmic dissipation.

The detailed morphology of the large-scale geomagnetic field and its rate of change is now well established thanks to 15 years of magnetic observations from low-Earth orbit satellites^16^. At the same time, there has been progress in core flow inversion techniques that now better account for unresolved small scales^17^,^18^,^19^, more fully incorporate information on the expected rotation-dominated structure of flows^17^,^20^ and include the effects of magnetic diffusion^21^,^22^. The resulting maps of flow within the core have highlighted the importance of a planetary-scale gyre^17^,^19^,^22^,^23^ consisting of generally equatorward flow around longitude 100° E, westward flow under the Atlantic hemisphere and generally poleward flow around 90° W (Fig. 2), that is remarkably persistent^19^,^22^,^24^ during the time interval for which core flows can be reliably determined.

Here, we describe the role played by this planetary gyre in historical geomagnetic dipole decay, and provide new estimates of the relative contributions of advection and magnetic diffusion to the dipole decay process. Changes in the Earth's dipole moment **m** are caused by changes in the electrical current density **J** within the core and hence, via Ampère's law, owing to changes of the magnetic flux density **B** within the core^7^


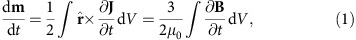


where *μ*_0_ is the magnetic permeability of free space. Working in spherical polar coordinates (*r*, *θ*, *φ*) and substituting from the magnetic induction equation, ∂**B**/∂*t*=∇ × (**u** × **B**)+*η*∇^2^**B**, where **u** is the fluid velocity and *η* is the magnetic diffusivity, taking the cylindrical axial component 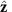 and re-arranging, the change in the Earth's axial dipole moment (ADM) may be written as^12^,^25^,^26^


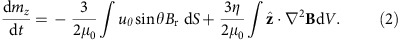


The first term on the right denotes the contribution from the meridional transport of flux by advection, while second describes the contribution from magnetic diffusion. By mapping −3/2*μ*_0_ *u*_*θ*_sin*θB*_r_, it is therefore possible to pinpoint locations where advective processes contribute most to axial dipole moment changes^12^,^26^.

## Results

### Simple illustrations of gyre-driven dipole decay

**Figure 1:**
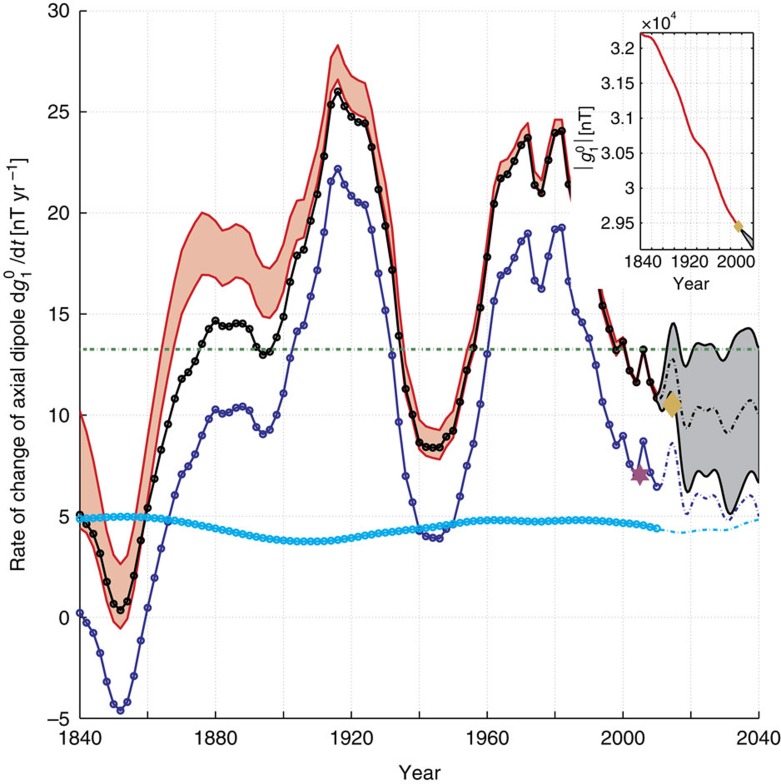
Observed and modelled decay of the geomagnetic axial dipole. Axial dipole magnitude 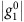 since 1840 (inset, red line, units: nT) and its rate of decay 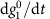 (red shaded area shows one standard deviation uncertainties, units: nT yr^−1^), from the COV-OBS^6^ geomagnetic field reconstruction. Comparable dipole decay rates are produced by a prototype gyre acting on an asymmetric field (green dot–dashed line, see also Fig. 2a), and by a more realistic filtered gyre flow, acting on the observed field averaged over 2000–2010 (purple star, see also Methods section and Fig. 2c). The solid black line with dots is the retrieved axial dipole decay rate from a series of 3D inversions for the field and flow within the core, based on geodynamo model multivariate statistics^22^ (see also Methods section and Figs 3 and 4). Each dot represents an independent inversion for the core state; these inversions are equally spaced in time. For the 3D inversion results, the dipole decay rate can be decomposed into its advective (dark blue line with dots) and diffusive (light blue line with dots) components. The grey area shows the 1 s.d. spread of an ensemble of 40 geodynamo model forward calculations, initialized using the estimated core state^22^ in 2010, with randomized realizations of small scales; the ensemble mean is shown by the black dot–dash line. Corresponding ensemble mean advective and diffusive contributions are given by the dark and light blue dot–dashed lines. The latest values for the axial dipole and its decay rate in 2014, as determined using the data from ESA's Swarm satellite constellation^39^, are marked by the gold diamonds.

**Figure 2:**
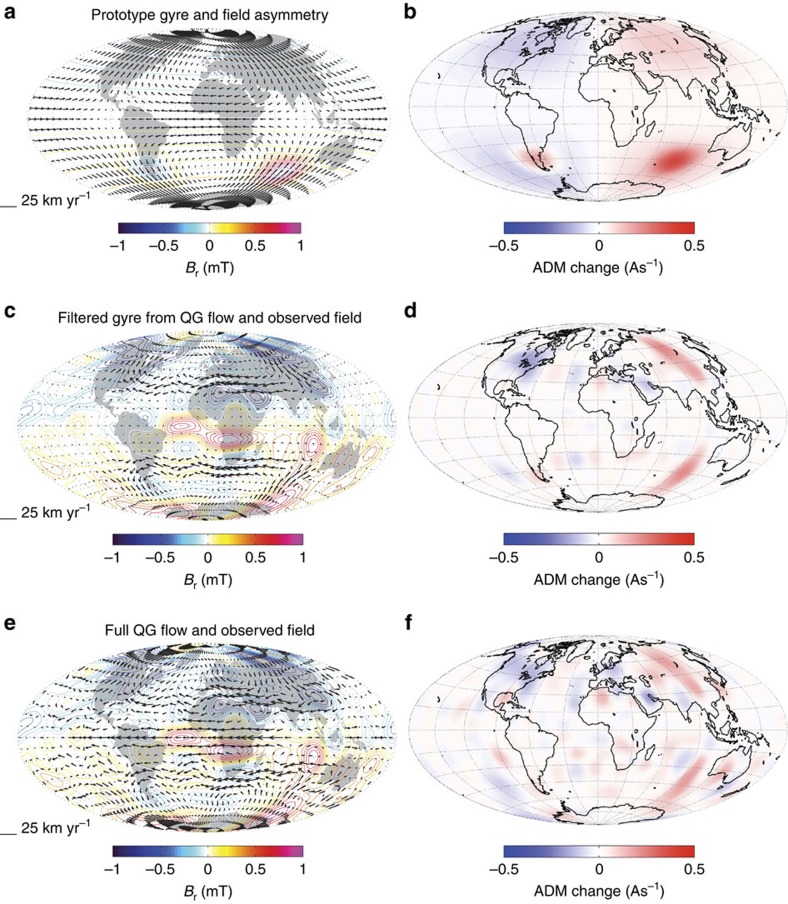
Gyre-driven dipole decay mechanism. Maps of the core surface showing (**a**) a prototype example of gyre-driven dipole decay, with a single core surface flow harmonic (arrows) acting on an axial dipole field with an imposed asymmetry in the southern hemisphere (reversed flux in the west and strong normal flux in the east); contours show the geometry of the imposed radial magnetic field *B*_r_ (units: mT) and (**b**) the associated map of advective contributions to axial dipole moment (ADM) change from core surface meridional flux transport −3/2*μ*_0_ *u*_*θ*_sin*θB*_r_ (units As^−1^) see equation (2). When integrated over the core surface, this gives the ADM change. Red indicates contributions to axial dipole decay, blue indicates contributions to axial dipole growth. (**c**) Here we see a more realistic case with a filtered gyre flow (arrows), extracted from an observation-based quasi-geostrophic core flow inversion^6^,^19^ (see **e**) acting on the known core surface field^6^ (contours), where both field and flow have been averaged over 2000–2010. (**d**) The associated map of meridional flux transport contributions to ADM change. (**e**,**f**) The same quantities for the full quasi-geostrophic core flow inversion^19^ which was filtered to obtain the flow in (**c**).

In Fig. 2a,b, we present a prototype example of our proposed gyre-driven mechanism for dipole decay. The essential ingredients are a departure of the field from axial symmetry and meridional flows that, on average, transport normal flux equatorward and reversed flux poleward. In Fig. 2a, starting with a negative axial dipole field as for the Earth today, this is achieved by placing strong normal field where there is equatorward flow and reversed field where there is poleward flow. Fig. 2b shows the resulting map of −3/2*μ*_0_ *u*_*θ*_sin*θB*_r_, which has a net positive value when integrated over the core surface, indicating the magnitude of the (negative) axial dipole is decaying. The maximum flow speed in this example is 19 km yr^−1^, the assumed magnitude of the axial dipole field at Earth's surface is −30,000 nT, the imposed radial field asymmetries are ±0.8 mT at the core surface, and the resulting rate of axial dipole decay is 13.6 nT yr^−1^ (green line, Fig. 1). Despite its simplicity, this demonstrates how a gyre with an Earth-like flow speed^27^, acting on a reasonable field asymmetry, can produce the observed magnitude of axial dipole decay.

Fig. 2c,d presents a more realistic scenario involving the known large-scale radial field at the core surface^6^, acted on by a recent observation-based quasi-geostrophic core flow^19^, that has been filtered to leave only the planetary gyre structure (see Methods section). Both this filtered gyre flow and the earlier prototype flow from Fig. 2a are equatorially symmetric, as required by the Taylor–Proudman theorem for rotation-dominated flows^17^. Both field and flow in Fig. 2c have been averaged over the decade 2000–2010 during which there are excellent observational constraints, thanks to the availability of magnetic data from the CHAMP and Øersted satellites and an extensive network of ground observatories. The integrated value of −3/2*μ*_0_ *u*_*θ*_sin*θB*_*r*_ mapped in Fig. 2d is again positive, so meridional flux transport once more causes dipole decay (purple star, Fig. 1). In this case there is little net contribution to dipole decay from the northern hemisphere, where intense normal flux is advected both poleward (under North America) and equatorward (under Asia). The dipole decay instead originates in the southern hemisphere, in agreement with the findings of previous observational studies^2^,^28^, due to the vigorous equatorward transport of intense normal flux south-west of Australia that is not balanced as there is a lack of intense normal flux (and presence of some reversed flux) in the region beneath South America where the flow is poleward. It is this asymmetry in the southern hemisphere magnetic field, that also results in the South Atlantic Anomaly^29^ at Earth's surface, which enables the gyre to drive the present dipole decay. Fluctuations of the meridional flow, particularly in the eastern equatorward limb of the gyre, can in this configuration easily generate rapid fluctuations in the dipole decay rate. Fig. 2e presents the quasi-geostrophic flow averaged over 2000–2010 without filtering; as shown in Fig. 2f, it produces similar patterns of meridional flux transport.

### Three-dimensional core state inversions

If meridional flux advection is capable of producing the observed rate of dipole decay, what then is the role of the magnetic diffusion that we have neglected in the above simple examples? Determining the role of diffusion in geomagnetic field evolution is challenging as it requires knowledge of the magnetic field structure within the core. In numerical geodynamo models^8^, the equations of conservation of momentum, magnetic induction and heat transport are time-stepped throughout the core, for prescribed values of control parameters. Since the magnetic fields and velocity fields are completely known, the role of magnetic diffusion can be fully assessed; whether or not the resulting kinematic processes are relevant to the Earth depends largely on the magnetic Reynolds number *R*_m_=*UL*/*η*, whether *U* is a typical velocity, *L* is a typical length scale and *η* is again the magnetic diffusivity; typical estimates^11^,^30^ for the Earth's core are in the range 1000–1500.

It has recently been demonstrated that multivariate statistics (linear correlations between fields) collected during a numerical dynamo forward calculation may be used as prior information in an inversion to estimate a complete field and flow state within the core that is consistent both with geomagnetic observations and that numerical dynamo^22^. We have examined a series of such inversions^22^ based on the COV-OBS^6^ geomagnetic field model and taking prior information from a specific numerical dynamo, hereafter referred to as the coupled earth or CE dynamo^31^, with a relatively large *R*_m_=942 that generates a planetary gyre similar to that indicated by the observations. Fig. 1 presents the axial dipole rate of change obtained from these three-dimensional (3D) inversions (black line with dots), including a decomposition into the respective advective (dark blue line with dots) and diffusive (light blue line with dots) contributions. The fluctuations in the observed rate of dipole decay are closely tracked by fluctuations of the advective component. The contribution of magnetic diffusion to dipole decay is on the other hand almost constant at about 5 nT yr^−1^. We conclude that meridional advection of flux is usually responsible for majority of the dipole decay, especially when the rate of decay is rapid. For example in 1980 more than 80% of the decay rate can be attributed to advective processes, with maps of −3/2*μ*_0_ *u*_*θ*_sin*θB*_*r*_ showing an enhanced contribution to dipole decay by very strong equatorward flux transport south-west of Australia. In addition to the decrease in the magnitude of the axial dipole over the past 170 years, the dipole tilt angle has also simultaneously decreased^29^, meaning that the equatorial dipole is decreasing even faster than the axial dipole; this is also likely to be a primarily advection-driven process^26^.

### Geodynamo model forward calculations

**Figure 3:**
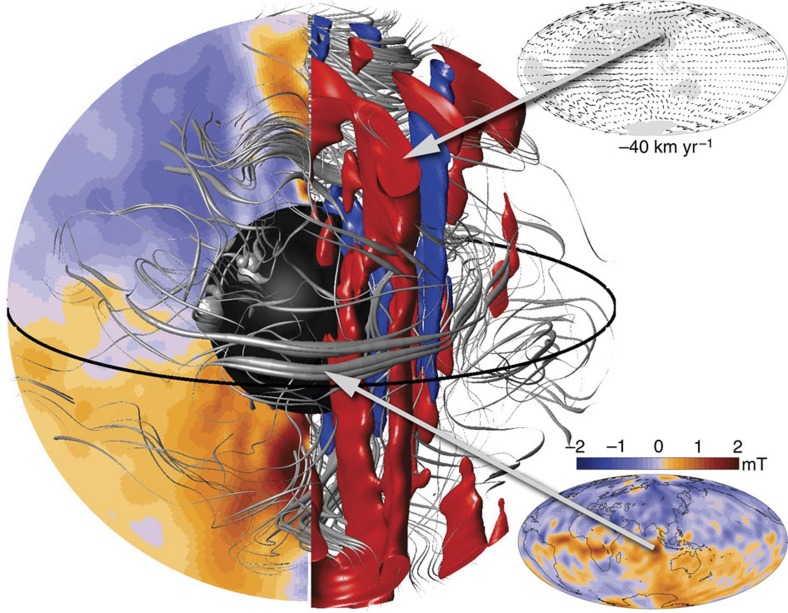
Estimated field and flow within the core in 2015. Volume visualization of the estimated magnetic field and flow within Earth's core in 2015 from a numerical geodynamo^31^ model forward run initialized with an inferred core state^22^ for 2010. Orange and blue contours show the intensity of the radial magnetic field, azimuthally averaged in a meridional plane within the shell, and at the core surface in the inset. The red and dark blue iso-surfaces are of constant axial flow velocity and illustrate intense columnar convection at the eastern meridional limb of the gyre, as also seen in the inset core surface flow plot. Field lines within the shell have thickness proportional to their magnetic energy. The inner core is black and the core–mantle boundary is transparent. The 3D view faces longitude 90° E, with a cutaway between 90° and 180° E.

In a further step, we started an ensemble of numerical dynamo forward runs starting from the inferred core state^22^ in 2010 (see Methods section). The resulting predictions are delimited by the grey region in Fig. 1 and show a continuing decay of the geomagnetic axial dipole. A visualization of an example of the 3D field and flow estimated within the core in 2015, from one of these dynamo forward calculations, is presented in Fig. 3. Strong equatorward flow in the eastern limb of the gyre at the core surface is seen to be connected with vigorous underlying columnar convection. The estimated magnetic field within the core is arranged into large-scale loops, with toroidal field in some locations being pushed out towards the core–mantle boundary. Flux expulsion therefore does take place in this dynamo, particularly at low latitudes, but it is not the dominant process driving the dipole decay or producing changes in the dipole decay rate.

**Figure 4:**
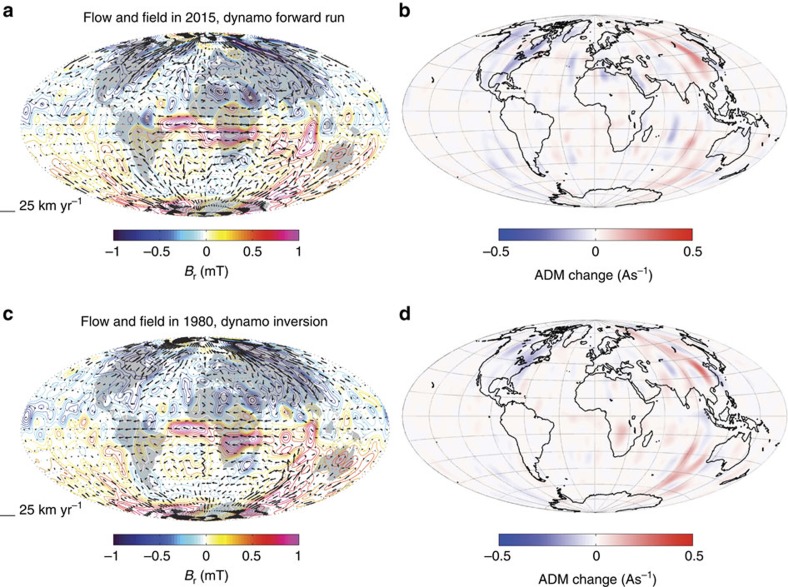
Gyre-driven dipole decay as inferred using the CE dynamo. Maps of the core surface showing (**a**,**c**) core surface flow (arrows) acting on the radial magnetic field *B*_r_ (units: mT) and (**b**,**d**) the associated maps of contributions to axial dipole moment (ADM) change from core surface meridional flux transport −3/2*μ*_0_ *u*_*θ*_sin*θB*_r_, units As^−1^. (**a**,**b**) Here the situation in 2015 is shown, for the same 3D state presented in Fig. 3, derived from a forward run of the CE dynamo model^31^ estimated from the inverted core state^22^ in 2010. (**c**,**d**) The same quantities for the inverted 3D core state in 1980 are shown, when the magnitude of dipole decay was twice as large as in 2015. Note that magnetic diffusion has been taken into account when deriving the flows presented here, which was not the case for the results presented in Fig. 2.

The core surface flow, radial magnetic field and the resulting advective contributions to ADM change, from the estimated core state in 2015 displayed in Fig. 3, are presented in Fig. 4a,b. Large-scale characteristics of this flow are found to be similar to those of the frozen-flux quasi-geostrophic flows of Fig. 2e, even though magnetic diffusion has now explicitly been taken into account. Furthermore, the map of the advective contributions to ADM change in Fig. 4b also shows similar major features to the maps obtained in our earlier simplified experiments (Fig. 2b,d,f), particularly in the eastern hemisphere. Comparing with maps of the estimated 3D core state^22^ in 1980 (Fig. 4c,d), we find that the advective contribution to dipole decay from the region south-west of Australia has notably decreased between 1980 and 2015, resulting in a decrease in the dipole decay rate from 24 to 10.5 nT yr^−1^. Examining the ensemble of dynamo forward calculations shown in Fig. 1, we find a continuing decrease in the dipole decay rate between 2010 and 2040.

### Analysis of the dipole decay process

In all the presented examples, the vast majority of positive and negative contributions from meridional flux advection to ADM change cancel on integration over the core surface; it is a small unbalanced remainder that is responsible for driving the dipole decay. In 1980, the ratio of 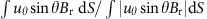 from the map in Fig. 4d is 0.17 indicating that, even in this case of relatively strong dipole decay, there was a rather large degree of cancellation in the meridional flux transport. The same ratio calculated from the map in Fig. 4b for 2015 is only 0.04 making the origin of the unbalanced contribution difficult to diagnose. One consequence of this finely balanced situation is that even minor changes in the meridional flux transport can cause relatively large changes in the dipole decay rate. The advective mechanism for dipole decay is certainly more clearly seen at times when the dipole decay rate is large, such as in 1980 (see Fig. 4c,d) when a large-scale imbalance is evident. On the other hand, the small length-scale structure of *B*_r_ and *u*_*θ*_ apparently play a more important role when the dipole decay rate is smaller (see Fig. 4a,b).

An alternative perspective on the origin of the dipole decay comes from examining the evolution of the magnetic energy per spherical harmonic degree at the core surface. First considering the COV-OBS^6^ field model, we find that between 1970 (when global satellite magnetic measurements were first available) and 2010, the percentage of the total energy (up to spherical harmonic degree 12) in the dipole field decreased from 45 to 42%, while the energy of the non-dipole core field increased from 55 to 58%, with the total energy remaining essentially constant. This is consistent with an advective transfer of energy from the dipole to the non-dipole field^32^. Since a decrease in the energy of some non-dipolar degrees (particularly 4 and 6) was also observed, it seems that the transfer of energy is not a simple forward cascade^33^. Turning to the CE dynamo forward runs initialized from the inverted core state^22^ in 2010, we also find an increase in the energy of the large-scale non-dipole magnetic field during dipole decay, lending further support to the hypothesis that the presently observed dipole decay is primarily an advection-driven process.

## Discussion

In a long, 130,000 years, forward run of the CE dynamo, we find that episodes of intense (20 nT yr^−1^ or greater) dipole decay are correlated to increased contributions from advective processes to the decay rate. During this long forward run, diffusion on average contributes 3 nT yr^−1^ towards dipole decay but it varies only weakly (standard deviation 2.9 nT yr^−1^). On the other hand, advective processes on average contribute 3 nT yr^−1^ to dipole growth (the CE dynamo is quasi-steady averaging over 130,000 years), but with a standard deviation of 6.1 nT yr^−1^, more than twice that of the diffusive processes. Fluctuations in meridional flux transport by advection are thus the most important kinematic mechanism for producing dipole growth and decay in the CE dynamo. On the other hand, we find no evidence for systematic increases in magnetic diffusion during the transient rapid dipole decay events exhibited by the model. It should, however, be remembered that the CE dynamo is designed to mimic the morphology of the present geomagnetic field and the historically observed patterns of secular variation, and not to study variations on the hundreds of kyr time scales relevant to reversals and excursions. Since it does not exhibit polarity reversals or sustained dipole collapse events, care is needed when interpreting the implications of our results for longer time scales. It remains possible that more dramatic events, not captured in the CE dynamo, may require a sustained increase in magnetic diffusion^15^,^34^. Despite these caveats, our results indicate that the presence of large-scale field asymmetries such as the South Atlantic Anomaly, together with fluctuations of meridional core flows, may turn out to be central to the time-dependent nature of the geodynamo. Issues of great interest for palaeomagnetic studies are now whether field asymmetries such as the South Atlantic Anomaly were always present during previous dipole decay episodes, and whether or not there is any evidence for the long-term persistence of the planetary gyre and its associated patterns of secular variation.

Our CE dynamo model assumes a well-mixed outer core. It has recently been argued that the outermost core may be stably stratified^35^, and that periodic, axisymmetric, flow oscillations of such a stratified layer may be responsible for fluctuations in the axial dipole^36^. The planetary gyre central to our proposed dipole decay mechanism is large scale and fairly steady, so it is expected to penetrate any such stratified layer^37^ and would in this scenario still produce dipole decay by the mechanism described above. In both the models, dipole variations result from fluctuations of the meridional flow at the core surface. The differences are that in our model the meridional flow variations are not periodic or axisymmetric (they are driven by convective fluctuations, especially in the eastern hemisphere) and that the zonal part of our flows naturally reproduce the observed decadal changes in the length of day^19^,^22^, whereas flow oscillations in a thin-stratified layer require additional coupling to unknown deeper flows.

Can the above insights shed any light on how long the present episode of dipole decay may continue? The necessary ingredients appear to be field asymmetry and meridional flows in appropriate locations. The South Atlantic Anomaly has been present throughout the era of direct geomagnetic observations (since 1840) and it continues to deepen^29^. Moreover, core flow inversions indicate that the planetary gyre and its meridional limbs have been rather stable over at least the past 175 years^19^,^22^,^24^. We therefore anticipate that the gyre will continue to drive geomagnetic dipole decay, at least for the next few decades. Going beyond this statement is presently difficult, as illustrated by the divergence of our ensemble of dynamo forward runs in Fig. 1. Better knowledge of small-scale fluctuations of the meridional flow, and their interactions with the small-scale magnetic field^28^,^38^, are necessary for improved prognostic models of the dipole decay process. High-quality magnetic observations now being collected by ESA's Swarm satellite constellation^39^, in combination with improved dynamic models and time-dependent data assimilation systems^40^, promise a more complete means of testing of these ideas.

## Methods

### Geomagnetic observations and field models

The geomagnetic dipole change between 1840 and 2010 is here taken from the COV-OBS time-dependent geomagnetic field model^6^. COV-OBS is derived from direct field measurements between 1840 and 2010 from ground observatories (annual means), collected by satellites (POGO, Magsat, Ørsted, SAC-C and CHAMP) and from ground surveys. The data compilation is the same as that previously used for the *gufm1* field model^3^ but with updated observatory and satellite data sets. COV-OBS was determined via a stochastic inversion, with a second-order auto-regressive stochastic process prior in time, permitting jerk events and time spectra in agreement with observatory data. The model includes spherical harmonics up to degree and order 14, and consists of both a mean model and second-order statistics in the form of a model covariance matrix. Fig.1 also presents the axial dipole and its rate of change in 2014 as determined from initial data collected by the Swarm satellite constellation^39^.

### Quasi-geostrophic core flows

The quasi-geostrophic core flow model presented in Fig. 2e was obtained by a frozen-flux inversion of the COV-OBS field model, with the flow restricted to an equatorially symmetric, columnar basis^19^. Time-correlated modelling errors owing to interactions between unresolved core surface motions and magnetic fields (from degree and order 15 to 30) were accounted for by recursive estimation of an ensemble of flows, updating at each iteration the covariance matrix for the flow coefficients. Quasi-geostrophic flows up to spherical harmonic degree and order 20 were produced at yearly intervals. Here, we used the time average between 2000 and 2010 of the ensemble average of these flows, up to degree and order 15. The filtered gyre flow of Fig. 2c was obtained by gridding this flow in physical space, removing flow from the polar regions, the Pacific region, the equatorial region and in the gyre centre and then projecting back onto the divergence-free poloidal–toroidal basis. The resulting filtered gyre flow was renormalized so the maximum amplitude of westward flow was identical before and after filtering.

### Geodynamo inverse and forward modelling

Estimates of the magnetic field and flow within the core were also derived from the COV-OBS field model, but utilizing *a priori* statistics from a 3D numerical dynamo simulation via the inverse geodynamo modelling procedure^20^,^22^. This involves first performing a stochastic inversion for the magnetic field throughout the core to spherical harmonic degree 30, from the COV-OBS poloidal field to degree 13, utilizing an a priori covariance matrix derived from a large collection of geodynamo model states^18^,^22^,^41^. Next, the core surface flow is inferred from the observed poloidal secular variation (again provided by COV-OBS), with diffusive effects included via the 3D core field estimated in the previous step^22^. Finally, the flow throughout the core is estimated by an additional stochastic inversion, again using a priori covariances from the geodynamo model states.

The numerical geodynamo model providing the prior information attempts to simulate as best as possible observed patterns of geomagnetic secular variation, in particular the westward drift and the Pacific–Atlantic dichotomy. Known as the CE dynamo model^31^, it solves for Boussinesq convection, buoyancy transport and magnetic induction in a spherical shell (inner/outer shell radii ratio 0.35) of electrically conducting liquid. This is coupled to a solid inner core with the same electrical conductivity and to an insulating solid outer spherical shell (mantle). Electrically conducting and no-slip boundary conditions are applied at the inner-core boundary. Electrically insulating and free-slip boundary conditions are applied at the core–mantle boundary. The mass anomaly flux at the inner-core boundary has a longitudinal hemispheric heterogeneity, which is maximum at longitude 90° E. while the core–mantle boundary has a heterogeneity motivated by lower-mantle seismic tomography^31^. The Ekman number Ek=*ν*/Ω*D*=3 × 10^−5^ (where *ν* is the fluid viscosity and *D* the fluid shell depth), the mass anomaly flux Rayleigh number is Ra=*g*_o_*f*/*ρ*Ω^3^*D*^2^=9.3 × 10^−5^ (where *g*_o_ is the gravity at the core–mantle boundary and *ρ* is the fluid density). The Prandtl and magnetic Prandtl ratios between the fluid viscosity, thermal and magnetic diffusivities *ν*, *κ*, *λ* are set to Pr=*ν*/*κ*=1 and Pr_m_=*ν*/*λ*=2.5. The numerical scheme involved a second-order finite differencing scheme in radius with 160 non-uniformly distributed points, and horizontally used spherical harmonics up to degree and order 133. Time-stepping was of second-order, semi-implicit type. The a priori statistics needed for the inverse geodynamo modelling were obtained from a model run of length half a magnetic diffusion time, where 800 complete state snapshots were stored at a spacing of 100 years.

Regarding the forward runs started in 2010, an ensemble of 40 states, each derived using the inverse geodynamo technique applied to the COV-OBS field model in 2010 (but with randomized realizations of the small-scale field and flow, compatible with the prior multivariate statistics) were used as the initial conditions for a series of short forward runs of the CE dynamo model.

## Additional information

**How to cite this article:** Finlay, C. C. *et al.* Gyre-driven decay of the Earth's magnetic dipole. *Nat. Commun.* 7:10422 doi: 10.1038/ncomms10422 (2016).
